# Enhancing Scheduling Performance for a Wafer Fabrication Factory: The Biobjective Slack-Diversifying Nonlinear Fluctuation-Smoothing Rule

**DOI:** 10.1155/2012/404806

**Published:** 2012-06-27

**Authors:** Toly Chen, Yu Cheng Wang

**Affiliations:** Department of Industrial Engineering and Systems Management, Feng Chia University, Taichung 40724, Taiwan

## Abstract

A biobjective slack-diversifying nonlinear fluctuation-smoothing rule (biSDNFS) is proposed in the present work to improve the scheduling performance of a wafer fabrication factory. This rule was derived from a one-factor bi-objective nonlinear fluctuation-smoothing rule (1f-biNFS) by dynamically maximizing the standard deviation of the slack, which has been shown to benefit scheduling performance by several previous studies. The efficacy of the biSDNFS was validated with a simulated case; evidence was found to support its effectiveness. We also suggested several directions in which it can be exploited in the future.

## 1. Introduction

Semiconductor manufacturing is undoubtedly one of the most noticeable high-technology industries because semiconductor products have widespread applications. However, the life cycles of new semiconductor products are getting shorter. Therefore, semiconductor manufacturers are facing pressure to meet the various needs of customers within shorter time spans. Manufacturers consider rapid product development, agile production, shortened response times, and similar strategies to be viable. All of these strategies compress the cycle times of related processes. Of the various types of cycle times, production cycle time is particularly important because it determines the time of delivery to customers. In other words, if the production cycle time is shortened, the delivery to customers will be faster. To this end, shortening the production cycle time through effective job dispatching is an important task [1]. Much research has been done concerning semiconductor shop floor control as a special type of supervisory control [2], particularly in the domains of deterministic scheduling and job dispatching. However, Chen and Lin [3], Chen and Wang [4], and Chen [5] have noted that for semiconductor factories, job dispatching is very difficult. Theoretically, this is an NP-hard problem. In practice, many semiconductor factories suffer from lengthy cycle times and thus are not able to make favorable promises to their customers.

This study discusses how to determine the sequence of jobs to be processed on each machine in a semiconductor factory so as to shorten the cycle times of jobs. To this end, an innovative dispatching rule is proposed, which involves the applications of fuzzy logic, artificial neural networks, and mathematical programming.

In this field, some innovative dispatching rules considering job parameters have been proposed recently. For example, Chen [6] reported a nonlinear fluctuation smoothing rule that uses the divisor operator instead of the subtraction operator, which diversifies the slack and makes the nonlinear fluctuation smoothing rule more responsive to changes in the parameters. Chen and Wang [7] also proved that the effects of parameters are balanced better by a nonlinear fluctuation smoothing rule than by a traditional one if the variation in the parameters is large. In short, magnifying the difference in the slack seems to improve scheduling performance. For these reasons, a biobjective slack-diversifying nonlinear fluctuation smoothing rule is presented in this study to improve the scheduling of job dispatching in a wafer fabrication factory. 

In a fluctuation smoothing rule, jobs that are expected to have long remaining cycle times are assigned lower slack values, which gives these jobs higher priorities to be processed and quickens their progress. There are two sorts of jobs with long remaining cycle times. The first sort comprises jobs that are just in their early stages; these jobs still have many stages to undergo. It is not necessary to deal with jobs of this type. The second type comprises jobs that have been delayed for long periods of time; these jobs have undergone few stages and have more unprocessed stages than the other jobs that started at the same time. Such a situation should be tackled somehow. However, even though these jobs have high slack values according to a fluctuation smoothing rule, they might not be assigned appropriately high priorities because sometimes many jobs have high slack values at the same time and we are not able to determine an absolute sorting for these jobs. To tackle this problem, we need a rule that is able to generate slack values that are as diverse as possible. To this end, we propose the biobjective slack-diversifying nonlinear fluctuation smoothing rule. This rule differs from 1f-biNFS because it maximizes the difference in the slack as measured by the standard deviation of the slack. There are many factors which must be optimized to achieve this goal, so a complex optimization problem must be solved to produce the rule. We apply a polynomial fitting technique to convert it into a more tractable form, for which several optimal solutions can be found. After screening some values from the specified range, the remaining values are used to construct an optimized 1f-biNFS rule.

The later sections of this paper are arranged in the following way.  Section 2 is dedicated to the literature review.  Section 3 provides the details of the proposed methodology. In Section 4, a simulated case is used to validate the effectiveness of the biobjective slack-diversifying nonlinear fluctuation smoothing rule. The performance levels of some existing rules in this field are also examined using the simulated data.  Section 5 concludes this paper and points out some interesting topics for future work.

## 2. Literature Review

Semiconductor manufacturing can be divided into four stages: wafer fabrication, wafer probing, packaging, and final testing. The most important and most time-consuming stage is wafer fabrication, which starts with approximately 25 wafers grouped as a lot. This lot is passed through hundreds of operations to build up complex layers of patterned metal and wafer materials that produce the required circuitry. In this study, we investigate job dispatching for this stage. Among the various categories of methods (including dispatching rules, heuristics, data mining-based approaches [8, 9], agent technologies ([8, 10–12], and simulation) in this field, dispatching rules (e.g., first-in first-out (FIFO), earliest due date (EDD), least slack (LS), shortest processing time (SPT), shortest remaining processing time (SRPT), critical ratio (CR), the fluctuation smoothing rule for the mean cycle time (FSMCT), the fluctuation smoothing rule for cycle time variation (FSVCT), least total work (LTWK), modified due date (MDD), operation due date (ODD), cost over time (COVERT), FIFO+, SRPT+, and SRPT++) have received a lot of attention these years [8–10] and are also the most prevalent method in practical applications. For the details of the traditional dispatching rules, refer to Lu et al. [13]. A recent simulation comparison is presented in Chiang and Fu [14].

Some advances in this field are introduced in the following. Altendorfer et al. [15] proposed the work in parallel queue (WIPQ) rule targeting at maximizing throughput at a low level of work in process (WIP). Zhang et al. [16] proposed the dynamic bottleneck detection (DBD) approach by classifying workstations into several categories and then applying different dispatching rules to these categories. Three dispatching rules including FIFO, the shortest processing time until the next bottleneck (SPNB), and CR were used. Depending on the current conditions in the wafer fabrication factory, Hsieh et al. [9] chose one approach from FSMCT, FSVCT, largest deviation first (LDF), one step ahead (OSA), and FIFO.

Chen [17] modified FSMCT and proposed the nonlinear FSMCT (NFSMCT) rule, in which he smoothed the fluctuation in the estimated remaining cycle time and balanced it with that of the release time or the mean release rate. To diversify the slack, the division operator was applied instead. Followed by Chen [18], the one-factor tailored NFSMCT (1f-TNFSMCT) rule and the one-factor tailored nonlinear FSVCT (1f-TNFSVCT) rule were proposed. Both rules contain an adjustable parameter in order to customize them for a target wafer fabrication factory. As a multiple-objective study, Chen et al. [19] proposed a biobjective nonlinear fluctuation smoothing rule with an adjustable factor (1f-biNFS) to optimize the average cycle time and cycle time variation at the same time. More degrees of freedom seem to be conducive to the performance of customizable rules. For this reason, Chen et al. [19] extended 1f-biNFS to a biobjective fluctuation smoothing rule with four adjustable factors (4f-biNFS). For a summary of these rules refer to Table 1. One drawback of them is that only static factors are used, and these factors need to be determined in advance. To this end, most studies (e.g., [17–19]) have performed extensive simulation. Such simulation is not only time consuming but it also fails to consider enough possible combinations of these factors. Chen [6] established a mechanism that was able to adjust factor values for 1f-biNFS dynamically (dynamic 1f-biNFS). However, even though satisfactory results were obtained in that experiment, there was no theoretical basis supporting the proposed mechanism. Chen [20] tried to relate the scheduling performance to the factor values with a back propagation network (BPN). Artificial neural networks have been widely applied to various control fields [21–23]. When such applications work, one can find the factor values that contribute to optimal scheduling performance. However, the explanatory ability of the BPN was not sufficient.

## 3. Methodology

The variables are defined as follows:

1. *R*_*i*_: the release time of job *i*, *i* = 1 ~ *N*,
2. BQ_*i*_: the total queue length before the bottlenecks at *R*_*i*_,
3. CT_*i*_: the cycle time (actual value) of job *i*,
4. *CTE*_*i*_: the estimated cycle time of job *i*,
5. *D*_*i*_^(*l*)^: the delay of the *l*th recently completed job at *R*_*i*_, *l* = 1 ~ 3,
6. FQ_*i*_: the total queue length in the whole factory at *R*_*i*_,
7. *Q*_*i*_: the total queue length on the processing route of job *i* at *R*_*i*_,
8. RCT_*ij*_: the remaining cycle time (actual value) of job *i* since step *j*,
9. RCTE_*ij*_: the estimated remaining cycle time of job *i* since step *j*,
10. SCT_*ij*_: the step cycle time (actual value) of job *i* until step *j*,
11. SCTE_*ij*_: the estimated step cycle time of job *i* until step *j*,
12. WIP_*i*_: the factory WIP at *R*_*i*_,
13. SK_*ij*_: the slack of job *i* at step *j*,
14. *U*_*i*_: the average factory utilization at *R*_*i*_,
15. *α*: max (*R*_*i*_) − min (*R*_*i*_),
16. *β*: max (RCTE_*ij*_) − min (RCTE_*ij*_),
17. *γ*: *N* − 1,
18. *λ*: the mean release rate.

Obviously,

(1) $$\begin{array}{c} \text{C}{\text{T}}_{i}=\text{SC}{\text{T}}_{ij}+\text{RC}{\text{T}}_{ij}. \end{array}$$

Replacing all variables with their estimates gives

(2) $$\begin{array}{c} \text{CT}{\text{E}}_{i}=\text{SCT}{\text{E}}_{ij}+\text{RCT}{\text{E}}_{ij}. \end{array}$$

### 3.1. Remaining Cycle Time Estimation

Before applying the biobjective slack-diversifying nonlinear fluctuation smoothing rule, the remaining cycle time required for each job must be estimated in advance. There is not a great deal of research in this field, but the fuzzy c-means (FCM) and fuzzy back propagation network (FBPN) approach of Chen et al. [24] has been shown to be effective [25–27] and therefore has been used in this study. In the FCM-FBPN approach, FCM is first used to cluster jobs with similar attributes. FCM performs classification by minimizing the following objective function: where *K* is the required number of categories; *n* is the number of jobs; *μ*_*i*(*k*)_ represents the membership of job *i* belonging to category *k*;  *e*_*i*(*k*)_ measures the distance from job *i* to the centroid of category *k*; *m* ∈ [1, *∞*) is a parameter to increase or decrease the fuzziness. The procedure of applying FCM to classify jobs is as follows.

(1) Establish an initial classification result.
(2) Iterations: obtain the centroid of each category as
  (4) $$\begin{array}{c} {\overset{-}{x}}_{\left(k\right)}=\left\{{\overset{-}{x}}_{(k)j}\right\},\\ {\overset{-}{x}}_{(k)j}=\frac{\sum_{i=1}^{n}\mu_{i\left(k\right)}^{m}x_{ij}}{\sum_{i=1}^{n}\mu_{i\left(k\right)}^{m}},\\ \mu_{i(k)}=\frac{1}{\sum_{l=1}^{K}{\left(e_{i(k)}/e_{i(l)}\right)}^{2/\left(m-1\right)}},\\ e_{i(k)}=\sqrt{\sum_{\text{all}\;j}{\left(x_{ij}-{\overset{-}{x}}_{(k)j}\right)}^{2}}, \end{array}$$
  where ${\overset{-}{x}}_{\left(k\right)}$ is the centroid of category *k* and *μ*_*i*(*k*)_^(*t*)^ is the membership of job *i* belonging to category *k* after the *t*th iteration.
(3) Remeasure the distance of each job to the centroid of every category, and then recalculate the corresponding membership.
(4) Stop if the following condition is satisfied. Otherwise, return to step (2):
  (5) $$\begin{array}{c} \underset{k}{\max\;}\;\underset{i}{\max\;}\left|\mu_{i\left(k\right)}^{\left(t\right)}-\mu_{i\left(k\right)}^{\left(t-1\right)}\right|<d, \end{array}$$

where *d* is a real number representing the threshold of membership convergence.

Finally, the separate distance test (*S* test) proposed by Xie and Beni [28] can be applied to determine the optimal number of categories *K*:

(6) $$\begin{array}{c} \mathrm{Min}\;\;S \end{array}$$

subject to

(7) $$\begin{array}{c} J_{m}=\sum_{k=1}^{K}\sum_{i=1}^{n}\mu_{i(k)}^{m}e_{i(k)}^{2},\\ e_{\min\;}^{2}=\underset{p\neq q}{\min\;}\left(\sum_{\text{all}\;j}{\left({\overset{-}{x}}_{(p)j}-{\overset{-}{x}}_{(q)j}\right)}^{2}\right),\\ S=\frac{J_{m}}{n\times e_{\min\;}^{2}},\\ K\in Z^{+}. \end{array}$$

The *K* value minimizing *S* determines the optimal number of categories.

The remaining cycle time of a job that is being processed in a wafer fabrication factory is the time still required to complete the job. If the job has just been released into the wafer fabrication factory, then the remaining cycle time of the job is its cycle time. The remaining cycle time is an important performance measure for all work-in-progress (WIP) in a wafer fabrication factory. To predict the remaining cycle time, we usually subtract the step cycle time from the cycle time forecast:

(8) $$\begin{array}{c} \text{RCT}{\text{E}}_{nj}=\text{CT}{\text{E}}_{nj}-\text{SC}{\text{T}}_{nj}. \end{array}$$

For this reason, we need to predict both the cycle time and the step cycle time.

After clustering, a portion of the jobs in each category is fed back into the FBPN as “training examples” in order to determine the parameter values for the category. The configuration of the FBPN is as follows.

**Figure 1:**
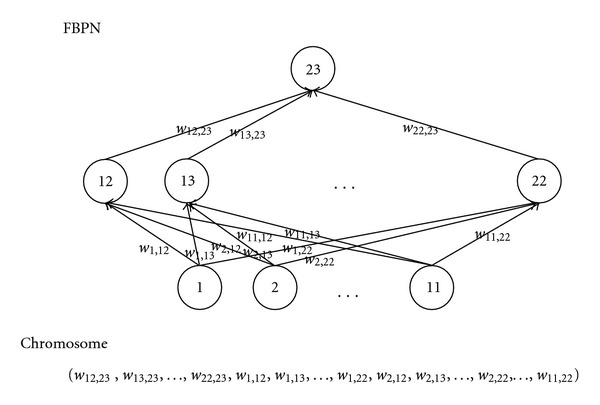
The chromosome used in the FCM-GA-FBPN.

(1) Inputs: eight parameters are associated with the *n*th example/job including *U*_*n*_, *Q*_*n*_, BQ_*n*_, FQ_*n*_, WIP_*n*_, and *D*_*n*_^(*i*)^(*i* = 1 ~ 3).
(2) There is a single hidden layer.
(3) The number of neurons in the hidden layer is the same as the number of neurons in the input layer.
(4) Output: the estimated (normalized) cycle time (CTE_*n*_) or estimated step cycle time (SCTE_*nj*_) of the example. In other words, there are two groups of BPNs. The first group estimates the CTE_*n*_'s of all the jobs to be scheduled, while the other group estimates their SCTE_*nj*_'s. The remaining cycle time estimate (RCTE_*nj*_) can be derived by subtracting SCTE_*nj*_ from CTE_*n*_.
(5) The network learning rule is the Delta rule.
(6) The transformation function is the Sigmoid function
  (9) $$\begin{array}{c} f\left(x\right)=\frac{1}{\left(1+e^{-x}\right)}. \end{array}$$
(7) The learning rate (*η*) ranges from 0.01 to 1.0.
(8) Initial conditions: because FBPNs tend to be very sensitive to initial conditions, in this study, a GA is employed to generate the initial values of the connection weights in the FBPN. Each chromosome is a vector of about 132 connection weights (see Figure 1). The connection weights are read off the FBPN and placed in a vector from left to right and from top to bottom. Each gene in the chromosome is a real number instead of a bit. To calculate the fitness of a given chromosome, the connection weights in the chromosome are assigned to the corresponding connections in the FBPN, the FBPN is trained using the training data, and the RMSE is returned. A low RSME value indicates high fitness:
  (10) $$\begin{array}{c} f=\frac{1000}{\text{RMSE}}. \end{array}$$
 An initial population of 100 vectors is chosen randomly, with each connection weight set to some uniformly distributed random value between −1.0 and +1.0. The mutation operator selects n noninput neurons and, for each incoming connection to those neurons, adds a uniformly distributed random value between −1.0 and +1.0 to the connection weight. The crossover operator takes two parent connection weight vectors; each noninput neuron in the offspring vector selects one of the parents randomly and copies the connection weights on the incoming connections from that parent to the offspring. Only one offspring is generated. In the child network, the weights of the incoming connections to neurons 12 and 23 come from parent 1, while those of the incoming connections to neurons 13 to 22 come from parent 2.
(9) Batch learning: the procedure for determining the parameter values is as follows. After preclassification, some of the adopted examples in each category are fed into the FBPN as “training examples” to determine the parameter values for the category. Two phases are involved at the training stage: the forward phase and the backward phase. In the forward phase, inputs are multiplied with weights, summed, and transferred to the hidden layer. Subsequently, activated signals are output from the hidden layer as
  (11) $$\begin{array}{c} {\tilde{h}}_{j}=\frac{1}{1+e^{-{\tilde{n}}_{j}^{h}}}, \end{array}$$
 where
  (12) $$\begin{array}{c} {\tilde{n}}_{j}^{h}={\tilde{I}}_{j}^{h}\left(-\right){\tilde{\theta }}_{j}^{h},\\ {\tilde{I}}_{j}^{h}=\sum_{\text{all}\;j}{\tilde{w}}_{ij}^{h}\left(\times \right){\tilde{x}}_{\left(i\right)}. \end{array}$$

${\tilde{h}}_{j}$ values are also transferred to the output layer with the same procedure. Finally, the output of the FBPN is generated as

(13) $$\begin{array}{c} \tilde{o}=\frac{1}{1+e^{-{\tilde{n}}^{o}}}, \end{array}$$

where

(14) $$\begin{array}{c} {\tilde{n}}^{o}={\tilde{I}}^{o}\left(-\right){\tilde{\theta }}^{o},\\ {\tilde{I}}^{o}=\sum_{\text{all}\;j}{\tilde{w}}_{j}^{o}\left(\times \right){\tilde{h}}_{j}. \end{array}$$

To improve the applicability of the FBPN and to facilitate comparisons with conventional techniques, the fuzzy-valued output $\tilde{o}$ is defuzzified according to the following formula:

(15) $$\begin{array}{c} d\left(\tilde{o}\right)=\int_{0}^{1}E\left(o^{\alpha }\right)d\alpha , \end{array}$$

where *o* ^*α*^ is the *α* cut of $\tilde{o}$. Then the output *o* is compared with the normalized actual cycle time (or step cycle time) *a*, for which the RMSE is calculated as

(16) $$\begin{array}{c} \text{RMSE}=\sqrt{\frac{\sum_{\text{all}\;\text{trained}\;\text{examples}}{\left(o-a\right)}^{2}}{\text{number}\;\text{of}\;\text{trained}\;\text{examples}}.} \end{array}$$

In the backward phase, the deviation between *o* and *a* is propagated backward, and the error terms of neurons in the output and hidden layers can be calculated, respectively, as

(17) $$\begin{array}{c} \delta^{o}=o\left(1-o\right)\left(a-o\right),\\ {\tilde{\delta }}_{j}^{h}={\tilde{h}}_{j}\left(\times \right)\left(1-{\tilde{h}}_{j}\right)\left(\times \right){\tilde{w}}_{j}^{o}\delta^{o}. \end{array}$$

Based on these error terms, adjustments to be made for connecting weights and thresholds can be obtained as

(18) $$\begin{array}{c} \Delta {\tilde{w}}_{j}^{o}=\eta \delta^{o}{\tilde{h}}_{j},\\ \Delta {\tilde{w}}_{ij}^{h}=\eta {\tilde{\delta }}_{j}^{h}\left(\times \right){\tilde{x}}_{i},\\ \Delta \theta^{o}=-\eta \delta^{o},\\ \Delta {\tilde{\theta }}_{j}^{h}=-\eta {\tilde{\delta }}_{j}^{h}. \end{array}$$

It is based on the basic gradient descent algorithm. For details refer to Chen [29] and Pendharkar [30]. To accelerate convergence, a momentum term can be added to the learning expressions. For example,

(19) $$\begin{array}{c} \Delta {\tilde{w}}_{j}^{o}=\eta \delta^{o}{\tilde{h}}_{j}+\alpha \left({\tilde{w}}_{j}^{o}\left(t\right)-{\tilde{w}}_{j}^{o}\left(t-1\right)\right). \end{array}$$

Theoretically, network learning stops when the RMSE falls below a prespecified level, or when the improvement in the RMSE becomes negligible over several epochs, or when a large number of epochs have already been run. Then test examples are fed into the FBPN and the accuracy of the network is measured with the RMSE. However, the accumulation of fuzziness during the training process continuously increases the lower bound, the upper bound, and the spread of the fuzzy-valued output $\tilde{o}$ (and those of many other fuzzy parameters); this might prevent the RMSE (calculated with the defuzzified output *o*) from converging to its minimal value. Conversely, network learning tends to shrink the centers of some fuzzy parameters. A fuzzy parameter can become invalid if its lower bound is higher than its center. To deal with this problem, the lower and upper bounds of all fuzzy numbers in the FBPN will no longer be modified if the following index converges to a minimal value:

(20) α∑all examplesmin  ((o1−a)2,(o3−a)2)number  of  examples +(1−α)∑all examplesmax  ((o1−a)2,(o3−a)2)number  of  examples,                       0<α<1.

Finally, the FBPN can be applied to estimate the cycle time or the step cycle time of a new job. When a new job is released into the factory, the eight parameters associated with the new job are recorded. Then the FBPN is applied to estimate the cycle time or step cycle time of the new job.

### 3.2. The Bicriteria Slack-Diversifying Nonlinear Fluctuation Smoothing Rule

The bicriteria slack-diversifying nonlinear fluctuation smoothing rule is derived by diversifying the slack in the 1f-biNFS rule:

(21) SKij =(i/λ−1/λ)1−ξ(Ri−ℬ)ξ(RCTEij−𝒜)(N/λ−1/λ)1−ξ(max (Ri)−ℬ)ξ(𝒞−𝒜) =ai1−ξbiξcij =aicij(biai)ξ,

where, *𝒜* = min (RCTE_*ij*_),  *ℬ* = min (*R*_*i*_),  *𝒞* = max (RCTE_*ij*_)

(22) $$\begin{array}{c} a_{i}=\frac{\left(i/\lambda \right)-\left(1/\lambda \right)}{\left(N/\lambda \right)-\left(1/\lambda \right)},\\ b_{i}=\frac{R_{i}-\min\;\left(R_{i}\right)}{\max\;\left(R_{i}\right)-\min\;\left(R_{i}\right)},\\ c_{ij}=\frac{\text{RCT}{\text{E}}_{ij}-\min\;\left(\text{RCT}{\text{E}}_{ij}\right)}{\max\;\left(\text{RCT}{\text{E}}_{ij}\right)-\min\;\left(\text{RCT}{\text{E}}_{ij}\right)}. \end{array}$$

The following two theorems explain the theoretical properties of 1f-biNFS.

**Theorem 1**

1f-biNFS is more responsive than the traditional fluctuation smoothing rules to changes in *R*_*n*_ if RCTE_*nj*_ *R*_*n*_  [6].

**Theorem 2**

The effects of parameters are balanced better by 1f-biNFS than by the traditional fluctuation smoothing rules if RCTE_*nj*_ − min (RCTE_*nj*_) ≥ *R*_*n*_ − min (*R*_*n*_), that is, if the variation in RCTE_*nj*_ is greater than that in *R*_*n*_, which is a common phenomenon in a wafer fabrication factory [7].

However, (21) is difficult to deal with. For this reason, the following polynomial fitting technique is used to convert it into a more tractable form:

(23) $$\begin{array}{c} x^{\xi }≅\left(0.94+1.77\xi -2.45\xi^{2}\right)+\left(0.02-0.16\xi +1.01\xi^{2}\right)x. \end{array}$$

**Figure 2:**
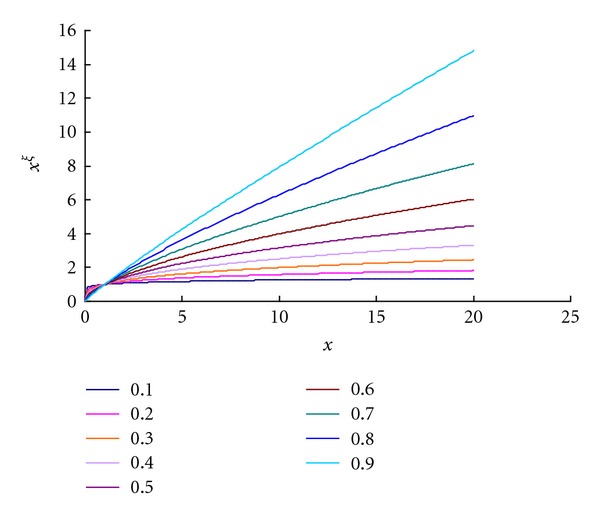
The linear approximation of *x* ^*ξ*^.

The mean absolute percentage error (RMSE) of (23) is less than 5% when *x* ≤ 20. The RMSE will not be a serious problem since it is the *ξ* value associated with the minimum *σ*_*SK*_*ij*__ to be found, not the SK_*ij*_ values. Such a polynomial fitting technique is especially effective when *x* exceeds 1 (see Figure 2). Applying (23) to (21) yields

(24) SKij ≅aicij(0.94+1.77ξ−2.45ξ2+(0.02−0.16ξ+1.01ξ2)biai) =(0.94aicij+0.02bicij)+(1.77aicij−0.16bicij)ξ  +(−2.45aicij+1.01bicij)ξ2 =dij+eijξ+fijξ2,

where

(25) $$\begin{array}{c} d_{ij}=0.94a_{i}c_{ij}+0.02b_{i}c_{ij},\\ e_{ij}=1.77a_{i}c_{ij}-0.16b_{i}c_{ij},\\ f_{ij}=-2.45a_{i}c_{ij}+1.01b_{i}c_{ij}. \end{array}$$

To diversify the slack, the standard deviation of the slack is to be maximized:

(26) σSKij=∑i=1N(SKij−SKj¯)2N−1=1γ∑i=1NSKij2−1N(∑i=1NSKij)2.

It is equivalent to maximizing the following term:

(27) ∑i=1NSKij2−1N(∑i=1NSKij)2 =∑i=1N(dij+eijξ+fijξ2)2−1N(∑i=1N(dij+eijξ+fijξ2))2 =∑i=1N(dij2+eij2ξ2+fij2ξ4+2dijeijξ+2dijfijξ2+2eijfijξ3)  −1N((∑i=1Ndij)2+(∑i=1Neij)2ξ2+(∑i=1Nfij)2ξ4      +2∑i=1Ndij∑i=1Neijξ+2∑i=1Ndij∑i=1Nfijξ2+2∑i=1Neij∑i=1Nfijξ3) =∑i=1Ndij2−1N(∑i=1Ndij)2+(2∑i=1Ndijeij−2N∑i=1Ndij∑i=1Neij)ξ  +(∑i=1Neij2−1N(∑i=1Neij)2+2∑i=1Ndijfij−2N∑i=1Ndij∑i=1Nfij)ξ2  +(2∑i=1Neijfij−2N∑i=1Neij∑i=1Nfij)ξ3  +(∑i=1Nfij2−1N(∑i=1Nfij)2)ξ4.

Taking the derivative of (27) with respect to *ξ*, and setting it equal to zero, we obtain

(28) (2∑i=1Ndijeij−2N∑i=1Ndij∑i=1Neij) +(2∑i=1Neij2−2N(∑i=1Neij)2+4∑i=1Ndijfij−4N∑i=1Ndij∑i=1Nfij)ξ +(6∑i=1Neijfij−6N∑i=1Neij∑i=1Nfij)ξ2 +(4∑i=1Nfij2−4N(∑i=1Nfij)2)ξ3 =w1+w2ξ+w3ξ2+w4ξ3=0,

where

(29) w1=2∑i=1Ndijeij−2N∑i=1Ndij∑i=1Neij,w2=2∑i=1Neij2−2N(∑i=1Neij)2+4∑i=1Ndijfij−4N∑i=1Ndij∑i=1Nfij,w3=6∑i=1Neijfij−6N∑i=1Neij∑i=1Nfij,w4=4∑i=1Nfij2−4N(∑i=1Nfij)2.

The optimal solution ξ* can be derived as

(30) ξ∗(1) =16w4(36w2w3w4−108w1w42−8w33+123w4      ×(4w23w4−w22w32−18w1w2w3w4         +27w12w42+4w1w33)1/2)1/3  −23(3w2w4−w32)/w4  /(36w2w3w4−108w1w42−8w33+123w4    ×(4w23w4−w22w32−18w1w2w3w4      +27w12w42+4w1w33)1/2)1/3−13w3w4,ξ∗(2) =−112w4(36w2w3w4−108w1w42−8w33+123w4       ×(4w23w4−w22w32−18w1w2w3w4          +27w12w42+4w1w33)1/2)1/3  +13(3w2w4−w32)/w4  /(36w2w3w4−108w1w42−8w33+123w4     ×(4w23w4−w22w32−18w1w2w3w4         +27w12w42+4w1w33)1/2)1/3−w33w4  +32(16w4(36w2w3w4−108w1w42−8w33       +123w4×(4w23w4−w22w32−18w1w2w3w4               +27w12w42+4w1w33)1/2)1/3    +23(3w2w4−w32)/w4    /(36w2w3w4−108w1w42−8w33+123w4     ×(4w23w4−w22w32−18w1w2w3w4       +27w12w42+4w1w33)1/2)1/3)i,

(31) ξ∗(3) =−112w4(36w2w3w4−108w1w42−8w33+123w4      ×(4w23w4−w22w32−18w1w2w3w4        +27w12w42+4w1w33)1/2)1/3  +13(3w2w4−w32)/w4  /(36w2w3w4−108w1w42−8w33      +123w4(4w23w4−w22w32−18w1w2w3w4             +27w12w42+4w1w33)1/2)1/3−w33w4  −32(16w4(36w2w3w4−108w1w42−8w33           +123w4(4w23w4                  −w22w32−18w1w2w3w4                  +27w12w42+4w1w33)1/2)1/3  +23(3w2w4−w32)/w4  /(36w2w3w4−108w1w42−8w33    +123w4(4w23w4−w22w32−18w1w2w3w4            +27w12w42+4w1w33)1/2)1/3)i.

However, not all of these terms are the answer since ξ* needs to satisfy the following constraints:

(32) $$\begin{array}{c} 0\leq \xi^{*}\leq 1 \end{array}$$

(33) $$\begin{array}{c} {\sigma_{\text{S}{\text{D}}_{ij}}^{\prime \prime }}_{}\left(\xi^{*}\right)=w_{2}+w_{3}\xi^{*}+w_{4}{\left(\xi^{*}\right)}^{2}\leq 0. \end{array}$$

Further, (31) and (32) are complex numbers that will only be considered if their imaginary parts are equal to zero. An example is given in Table 2 to illustrate the procedure mentioned previously. The optimal solution is ξ* = 0.94 with the maximum *σ*_SK_*ij*__ equal to 64115.3. Finally, ξ* can be used to construct an optimized 1f-biNFS as

(34) SKij=(βα(RCTEij−min  (RCTEij)))0.94·(Ri−RCTEij+0.94(RCTEij−min (Ri))).

However, it is possible that a job might have a very high or a very low slack value, which could distort the results. For this reason, we exclude the jobs with the highest or lowest slack value from (32):

(35) σSKij′=∑SKij≠Q(SKij−SKj¯)2N−3,=1γ−2∑SKij≠QSKij2−1N−2(∑SKij≠QSKij)2,

where *Q* = {max (SK_*lj*_), min (SK_*lj*_)}. As a result,

(36) w1=2∑SKij∉Qdijeij−2N−2∑SKij∉Qdij∑SKij∉Qeij,w2=2∑SKij∉Qeij2−2N−2(∑SKij∉Qeij)2+4∑SKij∉Qdijfij−4N−2∑SKij∉Qdij∑SKij∉Qfij,w3=6∑SKij∉Qeijfij−6N−2∑SKij∉Qeij∑SKij∉Qfij,w4=4∑SKij∉Qfij2−4N−2(∑SKij∉Qfij)2.

**Figure 3:**
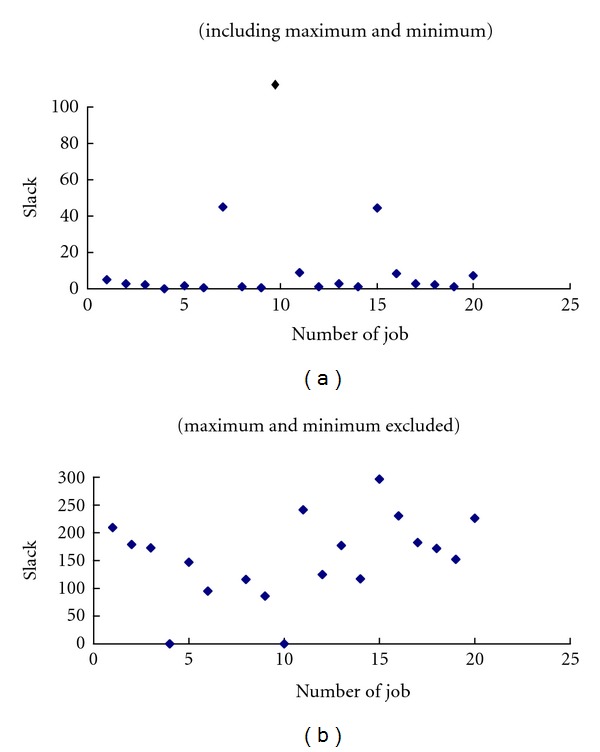
The effects of excluding the maximum and the minimum.

In the previous example, after excluding the minimum and maximum slack values, the optimal value of *ξ* was determined to be 0.11. We compared the results associated with the two settings in Figure 3. Obviously, the second setting achieved better slack diversification because it excluded the minimum and maximum slack values.

## 4. Experimental Results and Discussions

The effectiveness of the biobjective slack-diversifying nonlinear fluctuation smoothing rule was assessed with simulated data. To this end, a memory fabrication factory was simulated with a monthly capacity of up to 32,000 wafers. In the wafer fabrication factory, more than 500 workstations were devoted to single-wafer or batch production using 58 nm~110 nm technologies. The large-scale and the reentrant process flows made production control in the wafer fabrication factory a very tough task. The release policy was uniform; that is, jobs were released into it at a fixed interval, as is common in memory fabrication factories. FIFO was employed to sequence jobs on most of the workstations. The research sought to replace FIFO with better rules that might shorten the average cycle times and quicken deliveries to customers.

Although there were more than 10 products in the wafer fabrication factory, this research only considered the two major products that occupied most of the factory capacity; these were labeled A and B. The simulated jobs were assigned various priorities. Jobs with higher priorities were to be processed first. 

**Figure 4:**
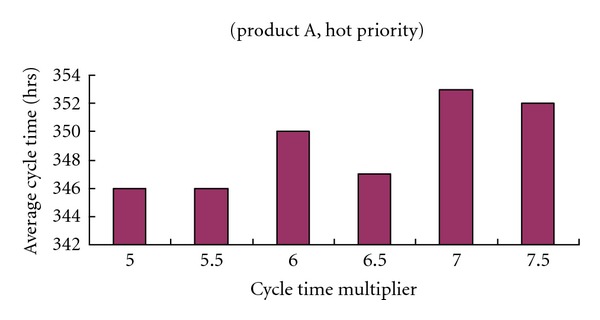
The effects of the cycle time multiplier on EDD.

**Figure 5:**
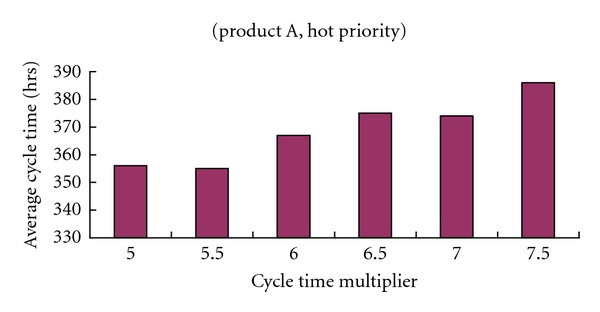
The effects of the cycle time multiplier on CR.

Nine existing approaches, FIFO, earliest due date (EDD), shortest remaining processing time (SRPT), CR, FSVCT, FSMCT, 1f-TNFSVCT, 1f-TNFSMCT, and 1f-biNFS, were evaluated for the simulated data. In EDD and CR, the internal due date of a job was determined by changing the cycle time multiplier [19]. Then, from several possible values, the value that gave the best performance was chosen (see Figures 4 and 5). Eleven values of *ξ* in 1f-TNFSMCT and 1f-TNFSVCT were taken from a list of possible values (0.1,0.2,…,1) and the *ξ*-value that returned the best schedule was taken as the output of the rule. The value of the factor in 1f-biNFS was determined in a similar way. The average cycle time, cycle time standard deviation of each product, and priority were compared for all approaches, as summarized in Tables 3 and 4.

1. Table 3 compares the performance levels of these methods with respect to the average cycle time. From the tabulated results, it is obvious that the biobjective slack-diversifying nonlinear fluctuation smoothing rule effectively shortened the average cycle times; for product B with normal priority, it was more than 10% better than FIFO. All the compared approaches were inferior to the biobjective slack-diversifying nonlinear fluctuation smoothing rule in this respect.
2. At the same time, it can be seen from Table 3 that the cycle time standard deviation was also controlled by applying the biobjective slack-diversifying nonlinear fluctuation smoothing rule. For a job of product A with the greatest time requirement and superhigh priority, the deviation of the cycle time from the average value was only 13 hours. This is remarkable for job dispatching in a wafer fabrication factory and conduces to reliable due date promises.
3. From Figures 4 and 5, it is obvious that the effects of the cycle time multiplier on EDD and CR were quite different, even though they employed the same method to determine the internal due date.
4. The biobjective slack-diversifying nonlinear fluctuation smoothing rule was better than the 1f-biNFS, with regard to both the average cycle time and the cycle time standard deviation. The advantages were 9% and 25% on average, respectively, which confirmed the usefulness of factor optimization to tailored rules like 1f-biNFS.

To determine whether the differences between the performance of the biobjective slack-diversifying nonlinear fluctuation smoothing rule and those of the nine existing approaches were significant, the following hypotheses were tested.

*H*_*a*0_: The shortening of the average cycle time of the biobjective slack-diversifying nonlinear fluctuation smoothing rule is the same as that of the compared existing approach.
*H*_*a*1_: The shortening of the average cycle time of the biobjective slack-diversifying nonlinear fluctuation smoothing rule is better than that of the compared existing approach.
*H*_*b*0_: The reduction in cycle time standard deviation of the biobjective slack-diversifying nonlinear fluctuation smoothing rule is the same as that of the compared existing approach.
*H*_*b*1_: The reduction in cycle time standard deviation of the biobjective slack-diversifying nonlinear fluctuation smoothing rule is better than that of the compared existing approach.

Several statistical methods have been developed for testing these hypotheses at a specified significance level *α*. One of the most commonly used methods is the Wilcoxon sign-rank test. The results are summarized in Table 5. The null hypothesis *H*_*a*_ was rejected at *α* = 0.025 or 0.05; the biobjective slack-diversifying nonlinear fluctuation smoothing rule was superior to six existing approaches in reducing the average cycle time. Furthermore, the advantage of the biobjective slack-diversifying nonlinear fluctuation smoothing rule over six existing dispatching rules in reducing cycle time standard deviation was also significant at *α* = 0.025.

## 5. Conclusions and Directions for Future Research

In this paper, we have presented a biobjective slack-diversifying nonlinear fluctuation smoothing rule modified from 1f-biNFS. Our new rule provides superior performance for job dispatching in a wafer fabrication factory. Our new rule maximizes the standard deviation of the slack dynamically; many studies have considered this feature to be conducive to scheduling performance.

A simulation experiment was set up to validate the effectiveness of the biobjective slack-diversifying nonlinear fluctuation smoothing rule.

1. The biobjective slack-diversifying nonlinear fluctuation smoothing rule incorporates the concept of factor optimization, so as to avoid the drawbacks of existing tailored nonlinear fluctuation smoothing rules. Through self-adjustment and continuous response to the changing conditions in the wafer fabrication factory, the biobjective slack-diversifying nonlinear fluctuation smoothing rule proved itself to be an effective dispatching rule in the simulation experiment.
2. The effectiveness of the biobjective slack-diversifying nonlinear fluctuation smoothing rule was fully revealed by the overall improvement in the scheduling performance, which was also examined and confirmed by statistical analyses.
3. The biobjective nature of the biobjective slack-diversifying nonlinear fluctuation smoothing rule was best revealed by the simultaneous improvements in the average cycle time and cycle time standard deviation.

Conversely, there are also disadvantages or limitations associated with the proposed methodology.

1. The way of diversifying the slack in the proposed methodology is subjective. For the same purpose, there are many other possible ways that can be tried to achieve better performance.
2. Compared with the existing dispatching rules, the proposed method requires more time to estimate the remaining cycle time and optimizing the rule content.

However, the same concept can be applied to optimize other rules to pursue better scheduling performance. This might be examined in future studies. In addition, to further evaluate the advantages and disadvantages of the proposed methodology, it has to be applied to a full-scale actual semiconductor factory.

## Figures and Tables

**Table 1:** The summary of some dispatching rules.

Rule name	Formula
1f-TNFSMCT	$\text{S}{\text{K}}_{ij}={\left(\frac{\beta }{\alpha \left(\text{RCT}{\text{E}}_{ij}-\min\;\;\left(\text{RCT}{\text{E}}_{ij}\right)\right)}\right)}^{\xi }\cdot (R_{i}-\text{RCT}{\text{E}}_{ij}+\xi (\text{RCT}{\text{E}}_{ij}-\min\;\;(R_{i})))$

1f-TNFSVCT	$\text{S}{\text{K}}_{ij}={\left(\frac{\beta \lambda }{\gamma (\text{RCT}{\text{E}}_{ij}-\min\;\;\left(\text{RCT}{\text{E}}_{ij}\right))}\right)}^{\xi }\cdot (\frac{i}{\lambda }-\text{RCT}{\text{E}}_{ij}+(\text{RCT}{\text{E}}_{ij}-\frac{1}{\lambda })\xi )$

1f-biNFS	$\text{S}{\text{K}}_{ij}=\frac{{\left(\left(i/\lambda \right)-\left(1/\lambda \right)\right)}^{1-\xi }{\left(R_{i}-\min\;\left(R_{i}\right)\right)}^{\xi }\left(\text{RCT}{\text{E}}_{ij}-\min\;\left(\text{RCT}{\text{E}}_{ij}\right)\right)}{{\left(\left(N/\lambda \right)-\left(1/\lambda \right)\right)}^{1-\xi }{\left(\max\;\left(R_{i}\right)-\min\;\left(R_{i}\right)\right)}^{\xi }\left(\max\;\left(\text{RCT}{\text{E}}_{ij}\right)-\min\;\left(\text{RCT}{\text{E}}_{ij}\right)\right)}$

4f-biNFS	SK_*ij*_ = (*R*_*i*_ − RCTE_*ij*_ + (RCTE_*ij*_ − min (*R*_*i*_)) · *f*_1_) · *α* ^−*f*_2_^
	$\cdot \left(\frac{i}{\lambda }-\text{RCT}{\text{E}}_{ij}+\left(\text{RCT}{\text{E}}_{ij}-\frac{1}{\lambda }\right)\cdot f_{3}\right)\cdot {\left(\frac{\gamma }{\lambda }\right)}^{-f_{4}}$
	$\cdot {\left(\frac{\text{(RCT}{\text{E}}_{ij}-\min\;\left(\text{RCT}{\text{E}}_{ij}\right))}{\beta }\right)}^{-(f_{2}+f_{4})}$

Dynamic 1f biNFS	$\text{S}{\text{K}}_{ij}=\frac{{\left(\left(i/\lambda \right)-\left(1/\lambda \right)\right)}^{1-\xi (t)}{\left(R_{i}-\min\;\left(R_{i}\right)\right)}^{\xi (t)}\left(\text{RCT}{\text{E}}_{ij}-\min\;\left(\text{RCT}{\text{E}}_{ij}\right)\right)}{{\left(\left(N/\lambda \right)-\left(1/\lambda \right)\right)}^{1-\xi }{\left(\max\;\left(R_{i}\right)-\min\;\left(R_{i}\right)\right)}^{\xi }\left(\max\;\left(\text{RCT}{\text{E}}_{ij}\right)-\min\;\left(\text{RCT}{\text{E}}_{ij}\right)\right)}$
	$\xi (t)=(\frac{1}{2})\left(\sin\left(\frac{\pi }{c}t\right)+1\right)$

**Table 2:** An example.

	*R*_*i*_	RCTE_*ij*_	SK_*ij*_	SK_*ij*_′
1	319	26	5.00	209.34
2	344	61	2.59	178.78
3	376	91	2.50	172.87
4	311	178	0.25	—
5	399	146	1.86	146.73
6	325	155	0.54	95.07
7	381	16	44.77	304.73
8	377	172	1.22	116.05
9	319	163	0.40	85.87
10	300	12	1.12*E* + 08	—
11	384	35	8.92	241.39
12	381	163	1.37	124.71
13	390	97	2.68	176.87
14	394	188	1.34	116.78
15	364	15	44.58	296.28
16	305	17	8.35	230.23
17	382	84	2.91	182.35
18	359	78	2.37	171.69
19	321	71	1.25	152.30
20	367	36	7.00	225.99

**Table 3:** The performances of various approaches in the average cycle time.

Avg. cycle time (hrs)	A (normal)	A (hot)	A (super hot)	B (normal)	B (hot)
FIFO	1256	401	320	1278	457
EDD	1087	346	306	1433	478
SRPT	966	350	309	1737	483
CR	1143	356	301	1497	470
FSMCT	1401	405	320	1408	430
FSVCT	1046	385	317	1745	519
1f-TNFSMCT	1353	379	298	1271	409
1f-TNFSVCT	1443	374	295	1326	398
1f-biNFS	1351	363	281	1285	413
The proposed methodology	1161	310	279	1145	394

**Table 4:** The performances of various approaches in cycle time standard deviation.

Cycle time std. dev. (hrs)	A (normal)	A (hot)	A (super hot)	B (normal)	B (hot)
FIFO	56	24	23	87	40
EDD	130	25	23	50	39
SRPT	246	32	23	106	30
CR	68	30	19	58	37
FSMCT	42	44	23	35	28
FSVCT	319	35	28	222	55
1f-TNFSMCT	81	43	22	49	25
1f-TNFSVCT	44	28	18	31	21
1f-biNFS	65	44	19	41	31
The proposed methodology	63	23	13	40	17

**Table 5:** The results of Wilcoxon sign-rank test.

	H_a0_	H_b0_
FIFO	*Z* = 2.02**	*Z* = 1.48
EDD	1.21	2.02**
SRPT	0.94	2.02**
CR	1.75*	2.02**
FSMCT	2.02**	0.67
FSVCT	1.21	2.02**
1f-TNFSMCT	2.02**	2.02**
1f-TNFSVCT	2.02**	−0.40
4f-biNFS	2.02**	2.02**

*P < 0.05, **P < 0.025, ***P < 0.01.
